# A modified BG-Sentinel trap equipped with FTA card as a novel tool for mosquito-borne disease surveillance: a field test for *flavivirus* detection

**DOI:** 10.1038/s41598-023-39857-1

**Published:** 2023-08-08

**Authors:** Sara Manzi, Luca Nelli, Claudia Fortuna, Francesco Severini, Luciano Toma, M. Di Luca, Alice Michelutti, Michela Bertola, Francesco Gradoni, Federica Toniolo, Sofia Sgubin, Florigio Lista, Michele Pazienza, Fabrizio Montarsi, Marco Pombi

**Affiliations:** 1 Dipartimento di Sanità Pubblica e Malattie Infettive, Sapienza Università di Roma, Rome, Italy; 2 School of Biodiversity, One Health and Veterinary Medicine, University of Glasgow, Glasgow, UK; 3 Dipartimento di Malattie Infettive, Istituto Superiore di Sanità, Rome, Italy; 4 Istituto Zooprofilattico Sperimentale Delle Venezie, Legnaro, Italy; 5 Istituto di Scienze Biomediche Della Difesa, Rome, Italy; 6 Stato Maggiore Della Difesa, Rome, Italy

**Keywords:** Ecological epidemiology, Viral infection, Animal behaviour, Epidemiology

## Abstract

Early detection of pathogens in vectors is important in preventing the spread of arboviral diseases, providing a timely indicator of pathogen circulation before outbreaks occur. However, entomological surveillance may face logistical constraints, such as maintaining the cold chain, and resource limitations, such as the field and laboratory workload of mosquito processing. We propose an FTA card-based trapping system that aims to simplify both field and laboratory phases of arbovirus surveillance. We modified a BG-Sentinel trap to include a mosquito collection chamber and a sugar feeding source through an FTA card soaked in a long-lasting viscous solution of honey and hydroxy-cellulose hydrogel. The FTA card ensures environmental preservation of nucleic acids, allowing continuous collection and feeding activity of specimens for several days and reducing the effort required for viral detection. We tested the trap prototype during two field seasons (2019 and 2021) in North-eastern Italy and compared it to CDC-CO_2_ trapping applied in West Nile and Usutu virus regional surveillance. Collections by the BG-FTA approach detected high species diversity, including *Culex pipiens*, *Aedes albopictus*, *Culex modestus*, *Anopheles maculipennis *sensu lato and *Ochlerotatus caspius*. When used for two-days sampling, the BG-FTA trap performed equally to CDC also for the WNV-major vector *Cx. pipiens*. The FTA cards detected both WNV and USUV, confirming the reliability of this novel approach to detect viral circulation in infectious mosquitoes. We recommend this surveillance approach as a particularly useful alternative in multi-target surveillance, for sampling in remote areas and in contexts characterized by high mosquito densities and diversity.

## Introduction

Mosquito-borne pathogens are spreading globally due to changes in various factors such as social, demographic, and environmental conditions, affecting their transmission patterns^1^. This may result in introduction of exotic pathogens in new areas, as well as re-emergence or intensification of transmission in endemic settings^2^.

In Europe, the increase of autochthonous cases of exotic mosquito-borne diseases highlights the vulnerability of this temperate region, as shown by dengue and chikungunya outbreaks reported in recent years^3–7^. Among endemic pathogens, West Nile virus (WNV) is widely present in many European countries^8,9^. The infectious cycle is zoonotic and involves *Culex pipiens* mosquitoes as main vector, several bird species as reservoir hosts, and humans and horses as dead-end hosts^10^. In 2018 and 2022, the two largest WNV transmission seasons occurred in central and southern Europe. Italy was the most affected country, with 576 human cases in 2018 and 586 in 2022^11^^,^^12^.

Another Flavivirus circulating in the European continent is Usutu (USUV), which belongs to the same serocomplex of WNV. It was detected for the first time in Europe in 1996 (Italy), and in the following years it spread among several European countries^13,14^. At present, at least 28 cases of USUV infection have been reported in humans^15,16^, with the first two cases of neuroinvasive infection worldwide described in 2009 in two immunocompromised patients in Italy^14,17^. Overall, current information suggests a potential public health importance of this zoonotic virus^18^.

The epidemiology of WNV and USUV varies among European countries due to climatic and environmental factors, leading to different surveillance strategies. However, European countries are increasingly adopting an integrated One Health approach, including human, veterinary and/or entomological surveillance^19^. In addition to WNV, the Community Epidemiological Surveillance Network includes chikungunya (CHIKV), dengue (DENV) and Zika (ZIKV) viruses in surveillance plan^20^. This decision has been supported by the evidence of epidemiological changes in arbovirus distributions and the introduction of invasive mosquito vectors^2^.

Early detection of pathogens in mosquito vectors is crucial in preventing outbreaks and has the potential to provide a timely indicator of pathogen circulation before spreading to vertebrate hosts and, therefore, to humans^21^. However, mosquito-based surveillance is expensive in terms of time, cost and labour, although far lower than the healthcare cost and the possible economic impact of an outbreak^22–26^. Vector-based surveillance involves collecting target species and transporting them to a laboratory for specimen processing and molecular analysis. In most cases, the cold chain must be maintained to preserve nucleic acid from degradation, which complicates sample handling and processing. Another difficulty in vector surveillance is that screening mosquito pools for pathogens can indicate infected samples, but not necessarily infectious ones, which can only be assessed by pathogen detection in saliva.

Recently, several studies investigated the exploitation of mosquito saliva or excreta in vector surveillance. These studies, adopting different trapping systems and sampling schemes, rely on the addition of solid substrates preserving nucleic acids (e.g. Whatman FTA card) to collect and preserve at environmental temperature the pathogens released from mosquitoes through saliva during sugar feeding or via their excreta during the captivity in the trap^27–41^. Indeed, the detection of pathogens from FTA cards offers many advantages compared to the screening of mosquito pools: absence of cold chain maintenance, decrease in working efforts and precise identification infectious specimens.

In this study, we propose a sampling approach based on a modified BG-sentinel trap equipped with an FTA card sugar feeding system, aimed at increasing mosquito survival^42^. We tested this approach during two sampling years (2019 and 2021) against the standard arbovirus surveillance sampling (CDC-CO_2_ trap) in an area endemic for West Nile and Usutu viruses (Veneto region, Italy), showing its effectiveness in arboviral detection.

## Methods

### Trapping device

**Figure 1:**
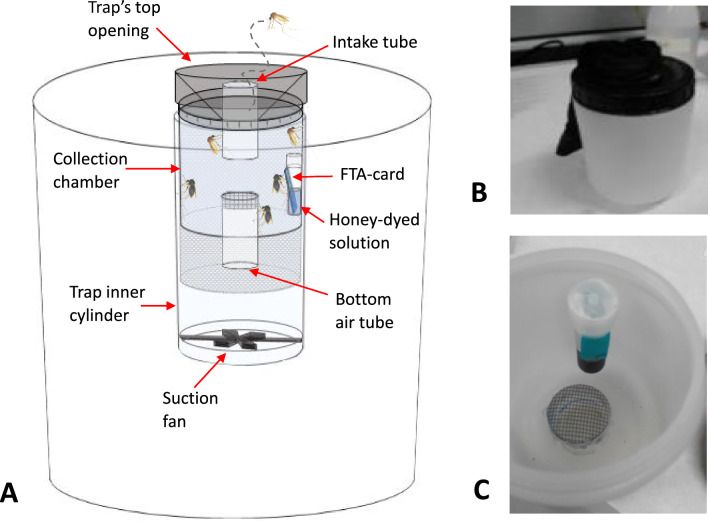
Modified BG-Sentinel trap used in this study. (**A**) Schematic representation of the trapping device. The feeding system comprises a collection chamber and a feeder. The collection chamber is a plastic container inserted into catch bag, with a top opening and a pipe system. The pipe system consists of a top intake tube that cuts across the container lid and an airflow tube at the bottom, covered with a fine mesh. A black nylon funnel covers the trap’s top opening and directs mosquitoes through the collection chamber’s intake tube; (**B**) Collection chamber; (**C**) Detail of the feeder showing the FTA card partially soaked in the sugar-dyed solution.

The trap used in this study was a modified BG-sentinel (Biogents AG, Regensburg, Germany, hereafter BG) equipped with a feeding system (Fig. 1) designed to keep mosquitoes alive longer. This system includes: (i) a collection chamber that provides a more comfortable environment than the original mesh bag, reducing stress and mortality of mosquitoes during trap activity; (ii) a pipe system that reduces airflow in the chamber, preventing rapid dehydration of mosquitoes; (iii) an FTA card sugar delivery system (feeder) through which collected mosquitoes can release pathogens during the sugar meal. The feeder is a plastic tube (height 6.5 cm, Ø1.5 cm; Euroclone S.p.A., Italy) filled with a honey-based solution composed by 2% hydroxy-ethyl-cellulose (average Mw 720000; Sigma-Aldrich, USA) in water and natural Acacia honey (Biscotti P. Gentilini S.r.l., Italy) in 3:2 proportion, in which a FTA Classic card (Whatman GE Healthcare, UK) is partially soaked. The solution is dyed with 0.15% methylene blue (a dye with low toxicity for mosquitoes; Merck KGaA, Germany) to allow the identification of sugar-fed mosquitoes. The feeder was preliminarily laboratory tested on *Aedes albopictus* mosquitoes to assess the sugar feeding rates (Supplementary File 1: Table S1).

### Collection sites and field sampling

**Figure 2:**
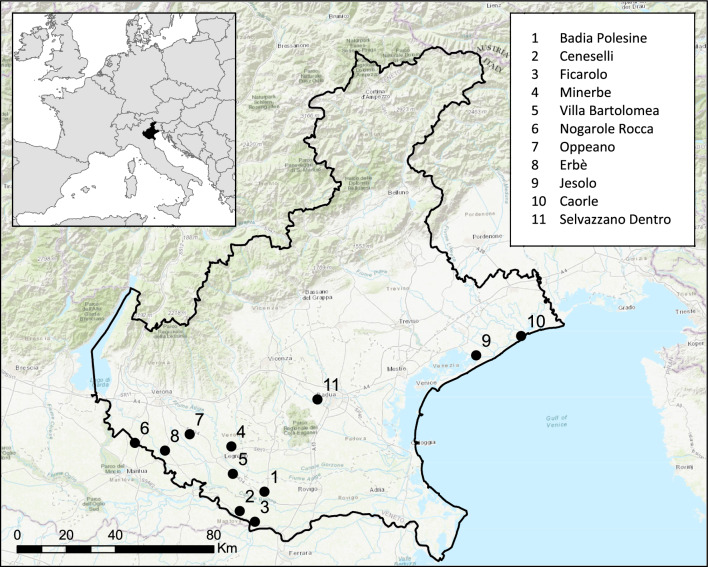
Map of the sampling sites in Veneto region. Points 1 to 10: sites sampled during 2019; point 11: sampling site of 2021. Background image source: OpenStreetMap. Image created using QGIS software (version 3.28; QGIS Development Team; 2022; QGIS Geographic Information System; Open Source Geospatial Foundation Project. https://qgis.org).

The field samplings were carried out in eleven municipalities of Veneto Region (North-eastern Italy, Fig. 2): Badia Polesine, Ceneselli, Ficarolo, Minerbe, Villa Bartolomea, Nogarole Rocca, Oppeano, Erbè, Jesolo, Caorle. In 2021, a single site in Selvazzano Dentro was sampled. All sites were characterized by both high vector density and endemic circulation of WNV and USUV, as reported from historical data obtained between 2010 and 2020 by the Istituto Zooprofilattico Sperimentale delle Venezie (IZSVe)^43^.

During 2019 sampling, four collections were carried out on alternate weeks in ten sites from July to August, resulting in a total of 112 observations. Our BG trap prototype was compared to a CDC-like trap (Italian Mosquito Trap; PeP, Cantu, Italy; hereafter CDC), which was chosen as a comparator because it is considered a highly effective device for *Culex pipiens* sampling in Italy^44^ and is the most used trap in WNV surveillance^45^. Both traps were provided with 2 kg of dry ice as a source of CO_2_. In addition, the BG trap was baited with BG-Lure (Biogents). The traps were deployed approximately 50 m from each other to avoid interference between them. The CDC trap was left active for about 24 h, while the BG trap was left active for an additional day, replacing dry ice and battery at the time of mosquito collection on the first day of sampling. Because the CDC traps utilized for WNV surveillance are consistently placed in the same location each year, it was not feasible to rotate the positions of the CDC and BG traps.

To investigate the performance of the BG trap over multiple working days in relation to mosquito infection prevalence, in 2021, only one site (Selvazzano Dentro) was tested (July–August) following a different experimental design. Five BG traps were left operational for four consecutive days every week, while a single CDC trap was used as a control, working for one night only, following the setup of the 2019 sampling. During the four-day collections of the BG traps, the CO_2_ was continuously supplied by a gas cylinder, and the fan was powered through power line. The mosquitoes were collected at the end of the fourth day.

### Mosquito identification and sample processing

The collected specimens were morphologically identified according to standard taxonomic keys^46^ and divided in pools of maximum 100 females, based on collection date, site, trapping method and species. In the first year of sampling (2019), the presence of blue dye was also assessed in all identified mosquitoes to determine the daily feeding rate for each species and predict the presence of saliva on the FTA card. All collected mosquitoes were identified while maintaining the cold chain and stored at − 80 °C. The FTA cards were individually placed into 2-ml Eppendorf tubes for at most 7 days at room temperature until following analysis. This time window was compatible with the detection of WNV according to preliminary tests performed under semi-field conditions (Supplementary File 1: Table S2, Figs. S1 and S2) and evidence from literature^47^. To avoid contamination, all cards were analysed separately from mosquitoes and on different days, sterilizing all the handling instruments after each sample manipulation.

### Viral RNA extraction from FTA cards and mosquitoes

Viral RNA from FTA cards was extracted using the QIAamp Viral RNA Kit (QIAGEN, Valencia, CA, USA). AVL buffer (Viral Lysis Buffer with carrier RNA, QIAGEN), EtOH (ITW Reagents, Italy) and RNA carrier amount were increased proportionally for a starting volume of 200 µl (800 µl AVL buffer, 8 µl RNA carrier, 800 µl EtOH). The samples were shaked for 2 h at room temperature after rehydration with AVL buffer and then the extraction proceeded according to the manufacturer’s protocol. RNA from pooled mosquitoes was extracted with an automated nucleic acid extraction system, to decrease hands-on time, increase sample throughput and reduce the risk of contamination. Before extraction, two 5 mm Tungsten Carbide Beads were added to each mosquito pool and the samples homogenized with the TissueLyser II (QIAGEN) at 30 Hz per 30’’ for two rounds. RNA was extracted from homogenate with an automatic extractor (Microlab STAR Hamilton, Americas, Australia & Pacific Rim) using MagMAX Pathogen RNA/DNA kit (Thermo Fisher Scientific, Waltham, MA, USA) following the high-volume manufacturer protocol.

### PCR protocols and sequencing for virus detection

RNA extracted from pooled mosquitoes and FTA cards was screened for the presence of flaviviruses using a RT-PCR, followed by hemi-nested PCR^48^ and sequencing. A SYBR Green-Based RT-PCR targeting 250 bp of the conserved region of the non-structural NS5 gene was performed in a final volume of 20 µl containing 10 µl of 2X QuantiNova SYBR Green RT-PCR Master mix (QIAGEN) (final concentration 1X), 0.25 µl of QuantiNova RT mix, 5.15 µl of RNase free water, 1 µl of 10 µM of MAMD forward primer (final concentration, 0.5 µM), 0.6 µl of 10 µM of cFD2 reverse primer (final concentration, 0.3 µM) and 3 µl of RNA template. The PCR thermal cycling was performed with Applied Biosystems StepOnePlus Real-Time PCR System (Thermo Fisher Scientific) for samples collected in 2019 and with MIC (BMS, Resnova, RM, Italy) for samples of 2021, as follows: initial incubation of 10 min at 50 °C and 2 min at 95 °C, amplification of 45 cycles at 95 °C for 5 s and 30 s at 60 °C, dissociation melting from 60 to 95 °C with a ramping rate of 0.3 °C/s. Analysis of the melting curve was carried out to determine the presence of viral RNA and the homogeneity of PCR products.

Positive results were confirmed with hemi-nested PCR and sequencing. PCR amplification was performed using FS788 e CFD2 primers^42^ targeting 220 bp of NS5 gene in a final volume 50 µl containing 5 µl of 10X Buffer II (AmpliTaq Gold DNA Polymerase with Buffer II and MgCl_2,_ Applied Biosystems, Thermo Fisher Scientific) (final concentration 1X), 4 µl of 25 mM MgCl_2_ (final concentration 2.0 mM), 1 µl of 10 mM dNTP (final concentration 0.2 mM), 2.5 µl of 10 µM of FS778 forward primer (final concentration 0.5 µM), 2.5 µl of 10 µM of CFD2 reverse primer (final concentration 0.5 µM), 5U of AmpliTaq Gold (final concentration 2.5U), 33.5 µl of ultrapure DEPC-pre-treated H_2_O and 1 µl of cDNA.

The PCR thermal cycling used for cFD2 and FS 778 primers were performed as follows: incubation of 10 min at 95 °C followed by 25 cycles of denaturation for 30 s at 94 °C, annealing at 54 °C for 30 s, extension at 72 °C for 30 s and final extension at 72 °C for 3 min.

The amplification products were identified by their molecular weights through electrophoresis in a 7% agarose gel stained with SYBR Gold Nucleic Acid Gel Stain 1X (Invitrogen, Thermo Fisher Scientific) and visualized under UV light using Gel Doc XR + Gel Documentation System (Bio-Rad, Hercules, California, USA). Positive PCR products were purified and sequenced in both directions using the same forward and reverse primers of heminested PCR, employing a 16-capillary ABI PRISM 3130xl Genetic Analyzer (Applied Biosystems, Foster City, CA, USA). Sequence data were assembled and edited with SeqScape software v2.5 (Applied Biosystems). Sequences obtained were aligned and compared with representative sequences available on GenBank database using Basic Local Alignment Search Tool (BLAST; http://blast.ncbi.nlm.nih.gov/Blast.cgi).

### Statistical analysis

Different generalised linear models (GLM) were used to investigate: (1) the relation between mosquito abundance observed with the BG with that observed with the CDC trap; (2) the variation of mosquito species diversity in relation to the trap; (3) the mosquito infection rate according to trap and working days. More specifically:

(1) To define the abundance of mosquitoes collected by the BG in relation to the CDC trap, we tested a GLM model with negative binomial distribution (to overcome data overdispersion). The mosquito abundance of the first day of collection was used as the response variable. The trap type, the Julian day and the sampling sites were included as explanatory variables. In addition, we included the working time as an offset term, to account for differences in the sampling effort for each collection. This model was run for the total female mosquito abundance and separately by species.

(2) To investigate the mosquito diversity, we calculated the Shannon diversity Index (SH) as:

$$\mathrm{SH }= \sum_{i=1}^{s}{p}_{i}\mathrm{ln}{p}_{i}$$

where p_i_ is the proportion of individuals of the ith species divided by the total number of individuals found in each collection and S is the species number. We then developed a GLM with a normal distribution, with SH as response variable and as covariates the same covariates that we used in model 1.

(3) To estimate the infection rate from pooled mosquitoes we calculated the maximum likelihood estimate (MLE) for each of detected virus, using the approach developed by CDC (https://github.com/CDCgov/PooledInfRate). We estimated the point and confidence interval of the MLE for each sampling year and for each detected virus, based on binary values samples of pooled specimens in relation to trap method, number of tested pools, size of tested mosquitoes per pool, and site (in case of 2019 sampling) or week (in case of 2021 sampling). We also estimated the MLE value for each year in relation to mosquito species and trap, including the number of tested pools and size of tested mosquitoes per pool.

All analyses were performed in the statistical environment R v.4.0.5^49^, using the following packages: PooledInfRate^50^, mass^51^, performance^52^, visreg^53^, ggplot2^54^, dplyr^55^ and reshape^56^.

## Results

### Mosquito abundance and distribution in the study area

**Figure 3:**
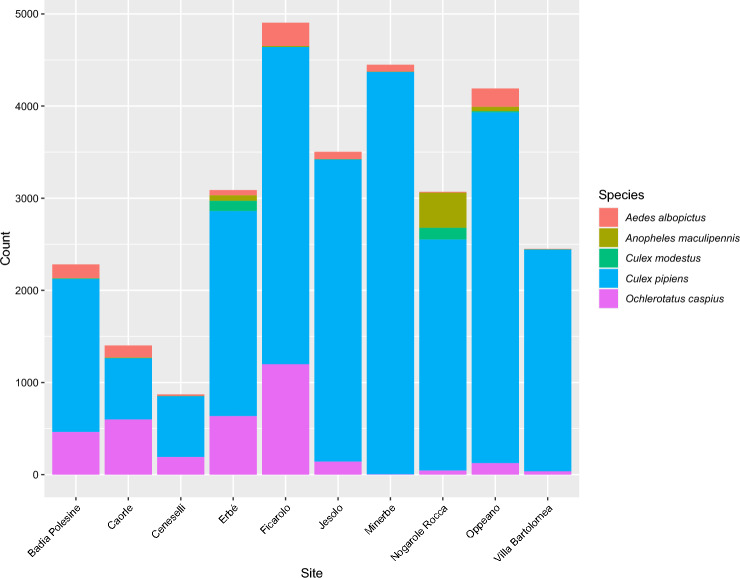
Abundance of female mosquitoes collected in 2019 in sampled sites of Veneto region. Data refers to female mosquito species collected with both trap types. Rare species are not reported (*Aedes vexans*, *Culiseta annulata*, *Coquillettidia richiardii*).

Overall, 40,944 (Female: 98.6%, Male: 1.4%) Culicidae specimens were collected during the 2019 sampling (Fig. 3), while 6,068 (Female: 98.3%, Male: 1.7%) were collected during 2021 sampling (overall, 98.4% of mosquitoes was successfully morphologically identified, BG: 99.9%, CDC: 96.6%).

**Figure 4:**
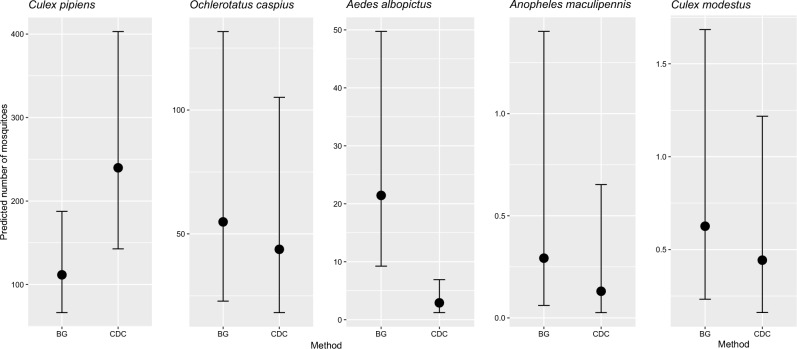
Effects of the trap method (BG or CDC) on the expected female mosquitoes collected per species, as resulting from the Generalized Linear Models. Data refers only to female mosquito species collected in the first day of sampling, when both traps were operating. Rare species are not reported (*Aedes vexans*, *Culiseta annulata*, *Coquillettidia richiardii*).

The CDC trap collected on average 2.16 times more *Cx. pipiens* female than BG (95% Confidence Interval: 1.568–2.941 females/trap), but 0.14 *Ae. albopictus* (95% CI 0.083–0.222 f/t) and 0.45 *An. maculipennis s.l.* (95% CI 0.231–0.862 f/t) specimens than those collected with BG (Fig. 4). Additionally, the BG and CDC traps showed similar performances in trapping *Oc. caspius* and *Cx. modestus*. The abundance of *Cx. pipiens* and *Cx. modestus* was also affected by the Julian day, with a significant decrease of the former species (0.96 f/t; 95% CI 0.946–0.965) and an increase of the latter (1.03 f/t; 95% CI 1.043–1.072). Differences in species abundances were observed among sites during 2019. Among the most abundant species, *Cx. pipiens* was significantly more abundant in Oppeano (2.25 f/t; 95% CI 1.115–4.541) and Minerbe (2.18f/t; 95% CI 1.076–4.395). *Aedes albopictus* was significantly more abundant in Oppeano (2.71 f/t; 95% CI 1.020–7.196), while it was less represented in Ceneselli (0.21 f/t; 95% CI 0.068–0.621), Nogarole Rocca (0.11 f/t; 95% CI 0.032–0.361) and Villa Bartolomea (0.10 f/t; 95% CI 0.030–0.357). *Ochlerotatus caspius* was significantly less abundant in Nogarole Rocca (0.11 f/t; 95% CI 0.036–0.331), Minerbe (0.01 f/t; 95% CI 0.001–0.033), Oppeano (0.32 f/t; 95% CI 0.109–0.938) and Villa Bartolomea (0.10 f/t; 95% CI 0.033–0.304) (Supplementary File 2).

### Mosquito species diversity in relation to trap type

Higher values of species diversity were observed in BG collection (SH: 0.73 95% CI 0.618–0.852) as compared to CDC (SH: 0.40 95% CI 0.317–0.488). Mosquito diversity increased significantly with the sampling dates, while significant differences among sites was observed, with lower mosquito diversity in Ceneselli, Minerbe and Villa Bartolomea as compared to other sampling sites (Supplementary File 2).

### Mosquito sugar-feeding rate in relation to species

**Figure 5:**
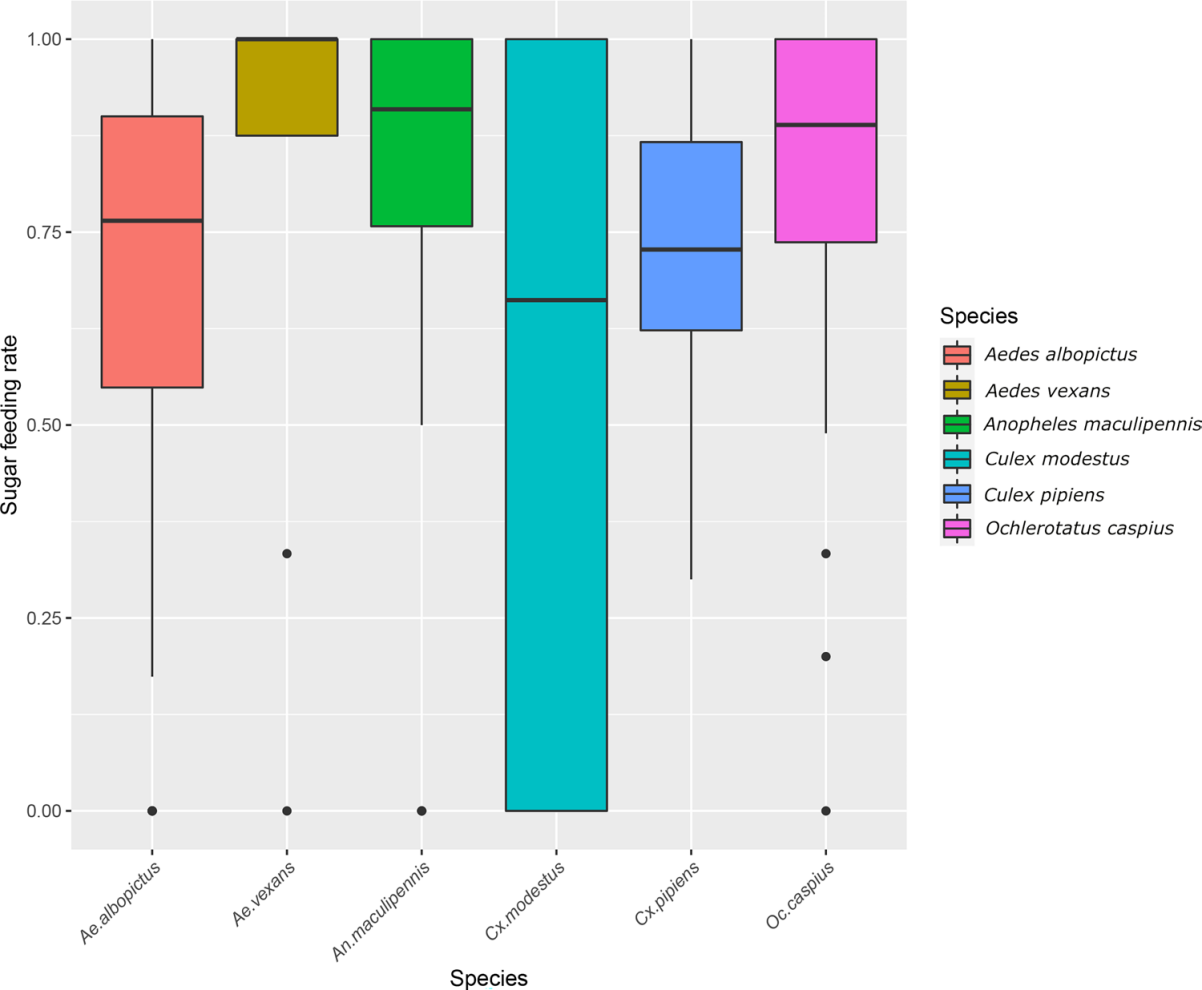
Sugar feeding rates on FTA cards for species collected with BG traps. Boxplots show minimum value (min), first quartile (Q1), median, third quartile (Q3) and maximum (max) value for *Aedes albopictus*, *Aedes vexans*, *Anopheles maculipennis s.l.*, *Culex modestus*, *Culex pipiens* and *Ochlerotatus caspius.*

High sugar feeding rates were observed in each collection (overall female mosquitoes; median: 80%; observation: 251; mosquitoes: 20,018). However, some variability was observed in relation to species, with a median frequency of 76% in *Ae. albopictus* (obs.: 63; mosq.: 1304), 100% in *Ae. vexans* (obs.:9; mosq.: 37), 91% in *An. maculipennis s.l.* (obs.:29; mosq.: 470), 66% in *Cx. modestus* (obs.: 20; mosq: 197), 73% in *Cx. pipiens* (obs.: 69; mosq.: 14,335), and 89% in *Oc. caspius* (obs.: 61; mosq.: 3675) (Fig. 5).

### Flavivirus detection from mosquitoes and FTA card

Mosquito pools and FTA cards positive to WNV and USUV are summarised in Tables 1 and 2. In 2019, changing the sampling effort of the BG traps from 24 to 48 h resulted in an increase of mosquito pools positive for WNV and USUV, from 2 and 3 to 5 and 10, respectively (out of 358 pools analysed). With this sampling effort, the BG trap achieved the same sensitivity of the CDC trap (5 pools WNV-2, 10 pools USUV, total 240 pools) (Table 1). In 2021, in the BG collection, where the traps were active for four consecutive days, 4 and 18 mosquito pools were positive to WNV-1 and USUV, respectively (out of 114 tested), while the CDC collection reported 2 out of 15 mosquito pools positive for USUV, only (Table 2).

**Table 1:** Positive samples to Flavivirus found in collection performed in 2019.

Year	Site	Method	Collected specimens*	Tested specimens	Pool + /N Pool	Virus Pool +	Species	MLE (95% CI) × 1000	Card + /N Card	Virus Card
2019	Badia Polesine (RO)	BG	1847	1805	1/36	WNV-2	*Cx. pipiens*	0.57 (0.032–2.735)	1**/8	WNV-2
		CDC	1455	1328	1/21	USUV	*Cx. pipiens*	0.76 (0.044–3.563)	NA	NA
	Ceneselli (RO)	BG	767	744	1/26	WNV-2	*Cx. pipiens*	1.43 (0.079–6.830)	0/8	NA
		CDC	559	509	1/12	WNV-2	*Cx. pipiens*	2.18 (0.118–10.300)	NA	NA
	Erbé (VR)	BG	2918	2833	3/47	USUV	*Cx. pipiens*	1.11 (0.289–2.959)	0/8	NA
					1/47	WNV-2	*Cx. pipiens*	0.36 (0.020–1.722)		
		CDC	1642	1591	2/27	USUV	*Cx. pipiens*	1.34 (0.235–4.349)	NA	NA
	Ficarolo (RO)	BG	5369	5189	1/67	USUV	*Oc. caspius*	0.19 (0.011–0.932)	0/8	NA
		CDC	1972	1878	1/25	WNV-2	*Cx. pipiens*	0.54 (0.031–2.593)	NA	NA
	Minerbe (VR)	BG	3191	3090	3/44	USUV	*Cx. pipiens*	1.02 (0.265–2.727)	0/8	NA
					2/44	WNV-2	*Cx.pipiens*	0.67 (0.180–2.168)		
		CDC	3143	2648	2/31	USUV	*Cx.pipiens*	0.79 (0.139–2.542)	NA	NA
					1/31	WNV-2	*Cx. pipiens*	0.38 (0.022–1.824)		
	Nogarole Rocca	CDC	2060	1791	1/26	WNV-2	*Cx. pipiens*	0.57 (0.032–2.721)	NA	NA
	Oppeano	BG	1798	1669	3/34	USUV	*Cx. pipiens*	1.97 (0.507–5.277)	1/8	USUV
		CDC	3453	2630	5/36	USUV	*Cx. pipiens (4),*	2.08 (0.766–4.523)	NA	NA
							*Oc. caspius (1)*			
					1/36	WNV-2	*Cx. pipiens*	0.39 (0.022–1.858)		

Collected specimens: total number of collected specimens (all species and both sexes) in sites with positive mosquito pools. Tested specimens: total number of tested female target species (*Aedes albopictus*, *Culex modestus*, *Culex pipiens* and *Ochlerotatus caspius*). N pool + /pool: number of positive pools/number of tested pools. Species: species of mosquitoes in positive pools. MLE (CI): estimated MLE value and 95% Confidence Interval of MLE value. N card + /N card: number of positive FTA cards/number of tested cards. *Data from BG refers to the sum of the 1st and 2nd sampling day, CDC refers to one day of sampling. **The positive FTA card and mosquitoes pools from Badia Polesine were detected in different traps.

**Table 2:** Positive samples to Flavivirus found in collection performed in 2021.

Year	Week	Method	Collected specimens	Tested specimens	Pool + /N Pool	Virus pool	Species	MLE (95% CI) × 1000	Card + /N Card	Virus Card
2021	30	BG	1278	1238	3/28	USUV	*Cx. pipiens*	2.71 (0.697–7.256)	0/5	NA
	31	BG	728	708	2/19	USUV	*Cx. pipiens* (1),	2.89 (0.532–8.757)	0/5	NA
							*Oc. caspius* (1)			
	32	BG	1067	1021	4/18	USUV	*Cx. pipiens*	4.82 (1.502–11.521)	1/5	USUV
	33	BG	1176	1150	4/24	USUV	*Cx. pipiens*	4.24 (1.314–10.273)	0/5	NA
					3/24	WNV-1	*Cx. pipiens*	3.01(0.760–8.131		
		CDC	163	159	1/3	USUV	*Cx. pipiens*	9.87 (0.425–49.063)	NA	NA
	34	BG	900	889	5/25	USUV	*Cx. pipiens*	6.86 (2.512–14.729)	1/5	USUV
					1/25	WNV-1	*Cx. pipiens*	1.19 (0.066–5.725)		
		CDC	254	253	1/4	USUV	*Cx. pipiens*	5.01 (0.251–23.309)	NA	NA

Collected specimens: total number of collected specimens (all species and both sexes) in week with positive mosquito pools. *Data from BG refers to four consecutive sampling days with five traps, CDC refers to one day of sampling. Tested specimens: total number of tested female target species (*Aedes albopictus*, *Culex modestus*, *Culex pipiens* and *Ochlerotatus caspius*). N pool + /pool: number of positive pools/number of tested pools. Species: species of mosquitoes in positive pools. MLE (CI): estimated MLE value and 95% Confidence Interval of MLE value. N card + /N card: number of positive FTA cards/number of tested cards.

Regarding the FTA cards, in 2019 one card was positive to WNV-2 and one to USUV (out of 72 tested), while in 2021 only two cards were positive for USUV (out of 25 tested). It should be noted that in Badia Polesine (2019 sampling), the WNV-2 positive FTA card was not associated with positive mosquitoes collected by the same trap (Tables 1 and 2).

In both sampling years, no *Ae. albopictus* were detected positive for WNV and USUV, or other flaviviruses (ZIKV, DENV), consistent with negative results from FTA cards.

The infection rate from pooled mosquitoes, resulted from MLE model, calculated for BG in 2019 for whole study area, ranged from 0 to 1.97 for USUV, and from 0 to 1.42 for WNV-2. In 2021, the MLE calculated for USUV and WNV-1 were 2.71–6.86 and 1.19–3.01, respectively.

## Discussion

### Modified BG trap vs CDC and feeding system performance

The modified BG trap was considerably more efficient than the CDC trap in collecting *Ae. albopictus* and *An. maculipennis* s.l. and showed similar performance with *Cx. modestus* and *Oc. caspius*, secondary vector species of WNV and USUV^57^. The BG trap also exhibited the best performance in terms of species diversity. These findings are consistent with results from different studies performed using standard BG-Sentinel traps^44,58–63^. The CDC trap was the best choice to target *Cx. pipiens* in our study, confirming literature data^44,63,64^. The lower performance of the BG trap in collecting *Cx. pipiens* could be overcome by using the trap for at least two consecutive days. However, other studies showed similar or better performance of the BG as compared to the CDC trap^60,65,66^, indicating the environment-dependence of the trapping systems that should be taken into account when a sampling scheme is set up in a new area.

A non-negligible limitation of the mosquito surveillance is the morphological quality of the collected specimens. In our study, the number of unidentified mosquitoes at species level significantly decreased when collected with the BG trap, due to the better sample preservation, which is an advisable property relevant for subsequent analysis, especially in arbovirus detection. This feature presumably stems from the high viability of trapped mosquitoes, in agreement with previous findings^42^. In an FTA card based system, the viability of mosquitoes in a trap working for several consecutive days increases the chances of sugar feeding of the trapped mosquitoes and, therefore, the release of pathogens on the FTA card. This is also a desirable feature in the perspective of deploying a surveillance system that does not require the rapid collection of mosquitoes to avoid degradation of viral RNA at field conditions.

The proper functioning of the feeding system of the BG trap presented here is confirmed by high sugar-feeding rates detected in the field (all species 80%, *Cx. pipiens* 73%, *Ae. albopictus* 76%), which has shown to be even higher than what observed in preliminary laboratory testing performed on *Ae. albopictus* (58%, Supplementary File 1: Table S1). This feeding rate is also consistent with other studies performed with different trapping systems^27,33,37,41^. Moreover, the feeding rate reported here could be probably underestimated, if compared to the actual number of mosquitoes that may have released saliva on the FTA cards. According to the literature, pathogens could be detected on the FTA card also in absence of a visible sugar meal^27,30,67^. This can be explained by several factors: (i) the mosquito probing is sufficient to release pathogens with saliva, although the sugar meal has not actually occurred; (ii) in the absence of laboratory dissection of specimens, the naked-eye detection of dyed mosquitoes might not be feasible, particularly in case of species with dark patterns; (iii) a partial sugar meal or an advanced digestion process may affect the colour of the dye, which becomes slightly visible or absent without dissection. Uncorrected estimates of sugar feeding rates could also be related to species collected. The differences observed between species in taking a sugar meal on the artificial support could be also linked to species-specific behaviours or physiological characteristics, such as a preferred sugary source and/or the time needed to be sugar-starved. This is in agreement with a laboratory study of Melanson et al.^67^, where 90% of tested *Ae. aegypti* took a sugar meal on the FTA card after 6 h, while in *An. stephensi* the observed feeding rate was only 37% after 24 h and 45% after 65 h, although both species were contemporarily sugar-deprived.

### Sensitivity of FTA card vs pooled mosquitoes in Flavivirus detection

The overall capability of the FTA card in our trapping system in detecting arbovirus was satisfactory, being able to detect the only known mosquito-borne viruses circulating in the study area (i.e., WNV and USUV). However, in our study the FTA card system showed a general lower sensitivity as compared to the pool mosquito analysis, which is in line with other literature evidence^30,68^.

Several factors may explain this phenomenon. For an infected mosquito to release a virus, the pathogen must first replicate in the vector after the blood meal and then disseminate throughout its body, reaching the salivary glands. Consequently, the viral titre in saliva is smaller as compared to the whole mosquito body, making it more difficult to be detected at low viral loads. It should also be noted that molecular virus detection in the whole mosquito carcass does not necessarily indicate mosquito infectivity. A positive FTA card can therefore act as an indicator of actually infective mosquitoes in a defined area, even if with a temporal delay compared to the standard method based on whole mosquito analysis. In fact, FTA cards detected USUV concurrently with highest MLE values, although the small number of positive events did not allow for statistical analysis. Indeed, in 2019, the USUV detection with FTA card was reported in a site with highest MLE value (MLE: 1.97), while in 2021 the positive FTA cards were observed in weeks with higher MLE values (MLE: 4.82, 6.86). This suggest that, with the sampling effort tested in this study, it was possible to detect USUV virus when its presence in vectors was high. For this reason, this strategy may be recommended only with the appropriate sampling effort, to compensate for the lower sensitivity of the FTA approach and be able to promptly react when the virus begins to circulate in mosquitoes. This is particularly true in the case of the integrated surveillance of WNV, where an early detection of viral circulation is mandatory to reduce the risk of inter-human transmission via blood, tissue and organ donation^69^. According to our results, two sampling days appear to be sufficient to obtain comparable numbers of positive mosquito pools with the BG (MLE: USUV = 0.51, WNV = 0.25) and the CDC trap (MLE: USUV = 0.62 WNV = 0.31). Nonetheless, during both sampling years, the FTA card failed to detect the virus in most positive traps. Increasing the number of BG traps in a site would have the double advantage to increase the likelihood to collect positive mosquitoes and the chance to detect pathogens on FTA cards. This is because: (1) if a collected mosquito is not starved yet, the increased captivity time will increase the chance sugar feeding; (2) if the mosquito viral load is low, more feeding attempts could release more virus on the FTA card; (3) if a recently infected mosquito remains alive in the trap for a few days, the virus replication will continue, becoming finally detectable in the saliva.

Among the flavivirus circulating in the sampled area, a higher prevalence of USUV compared to WNV was observed in both sampling years.

Regarding WNV lineages, the results from FTA cards did not completely overlap with those with mosquito pools. In fact, in 2019, both FTA cards and mosquito pools were found positive for WNV-2, while in 2021 WNV-1 was the only lineage found (in mosquito pools but not in FTA cards). Interestingly, WNV-1 was the only lineage reported in Italy from 1998 to 2011^70,71^, and was then largely replaced by the introduction of WNV-2^71–74^. The last record of WNV-1 in a mosquito pool was in Piacenza province in 2017, and this is the first evidence of a newly introduced strain in North-Eastern Italy since then^75^. This WNV-1 lineage showed highest similarity with the genome of a WNV-1 isolate identified in 2015 in the Camargue region (France)^76^, indicating the capacity of the BG-FTA approach to detect a newly introduced arboviral strain in the sampled area.

## Conclusion

In this study, we demonstrated that a trapping system based on a BG-sentinel trap modified to carry an FTA card can be an efficient tool for surveillance of mosquito-borne pathogens by exploiting mosquito sugar-feeding behaviour. This modified trap showed reliable performance in collecting mosquitoes belonging to several vector species (*Cx. pipiens, Ae. albopictus, Cx. modestus, An. maculipennis s.l.* and *Oc. caspius*), with good rates of sugar feeding observed in all species.

This approach may have some limitations in a low mosquito density context, particularly in areas of potential pathogen introduction. In contexts such as these, the BG-Sentinel with FTA card may lack sufficient sensitivity as an early warning system, where the occasional finding of infectious mosquitoes in a new area might remain undetected.

Although the sensitivity of the FTA card is lower than that of mosquito pool analysis, this method could be advantageous in many arbovirus surveillance contexts due to the long-term storage of RNA in field conditions and the reduced effort in sample handling and analysis. In particular, the BG-Sentinel with FTA card could be advantageous in absence of adequate tools for conservation and analysis of mosquitoes in loco (e.g., when maintaining a cold chain is not possible), which is particularly desirable in remote areas (where the necessity of a power supply for trap functioning could be solved with a solar panel). Additionally, this approach can be useful in contexts where the workload required to process high mosquito numbers in a short time is too high (e.g., in large areas or with high mosquito densities), as well as in the rapid detection of mosquito-borne pathogens in critical points at risk of new pathogen introductions (e.g., ports, train stations, airports etc.).

Finally, this surveillance system, which performed well in an endemic area for WNV and USUV, it could be particularly useful for arboviruses transmitted by *Ae. albopictus* and *Ae. aegypti*, species for which the BG-Sentinel trap is a very efficient trapping tool^58,59,62,77^. Given the outbreaks of CHIKV and DENV occurred in Europe in the recent past^3–6,78^, the proposed approach has the potential to be highly effective in a “multi-target” surveillance perspective, both in endemic tropical areas and in temperate contexts at risk of *Aedes*-transmitted virus outbreaks.

### Supplementary Information

Supplementary Information 1.

Supplementary Information 2.

## Data Availability

Data supporting the conclusions of this article are included within the article and its additional files. The datasets used and analysed during the present study are available from the corresponding author upon reasonable request.
