# Supplementary material for: A modified BG-Sentinel trap equipped with FTA card as a novel tool for mosquito-borne disease surveillance: a field test for flavivirus detection
Source: Sci Rep. 2023 Aug 8;13:12840. doi: 10.1038/s41598-023-39857-1 (PMC10409816; doi:10.1038/s41598-023-39857-1)
Supplement: Supplementary file 1 — Supplementary Information 1. [file 41598_2023_39857_MOESM1_ESM.docx]

**Supplementary file 1**

**S1a. Feeding test**

The feeding system was tested on *Aedes albopictus* in cages and BG traps placed in insectary (14:10 light-dark; 70±10% RH; 26±2°C), to assess the mosquitoes’ ability to take a sugar meal and estimate the sugar feeding rate on the FTA cards. Methylene blue, a dye with low toxicity for mosquitoes, was added to the sugar solution to identify fed specimens after feeding on the FTA card. To allow a gradual release of solution over the time, hydroxy-ethyl cellulose (HEC), a colloidal mixture of polymers with the feature of absorbing large quantities of water (over 90-95%), was also added. Under insectary conditions all the FTA cards inserted in feeders remained moist for over a week, reducing the volume of solution up to 50% without drying.

Different sugar delivery systems were tested in BG traps (Tab. S1):

- 0.5cm height x 6cm ∅ petri dish (VWR International S.r.l., Italy), containing 4.7ml of solution;
- 1cm height x 2cm ∅ PE-HD stopper, containing 1.6ml of solution;
- 1.7cm x 12cm 15ml Falcon tube (15ml, Thermo Fisher Scientific Inc., USA) with sponge, containing 5ml of solution;
- 1.7cm x 12cm Falcon tube (15ml, Thermo Fisher Scientific Inc., USA), containing 5ml of solution;

Sugar solutions (honey/30% sucrose with HEC -see main manuscript for manufacturer details- at 2%, 5%, 8%, 10% at the following proportions: 1:1, 1:3, 3:2).

The highest feeding rate in BG trap (58%) was achieved with FTA-card partially soaked in a solution based on honey and 2% HEC in 3:2 proportion, inserted in a 15ml tube.

| Delivery system/solution | N mosquitoes | mean % fed | N tests |
| --- | --- | --- | --- |
| ∅ 6cm petri dish with filter paper | **778** | **40%** | **27** |
| Cage | **778** | **40%** | **27** |
| 8% HEC + Honey (1:1) | 62 | 0% | 3 |
| 10% HEC + 30% Honey (1:1) | 41 | 5% | 2 |
| 10% HEC + Honey (1:3) | 427 | 12% | 11 |
| 5 % HEC + Honey (3:2) | 248 | 86% | 11 |
| ∅ 6cm petri dish with FTA-card | **71** | **21%** | **4** |
| Cage | **34** | **43%** | **2** |
| 8% HEC + Honey (3:2) | 34 | 43% | 2 |
| BGS | **37** | **0%** | **2** |
| 5% HEC + Honey (3:2) | 19 | 0% | 1 |
| 8% HEC + Honey (3:2) | 18 | 0% | 1 |
| ∅ 2cm stopper with FTA-card | **120** | **12%** | **8** |
| Cage | **20** | **9%** | **2** |
| 2% HEC + 30% sucrose | 9 | 0% | 1 |
| 2% HEC + Honey (3:2) | 11 | 18% | 1 |
| BGS | **100** | **13%** | **6** |
| 5% HEC + Honey (3:2) | 89 | 1% | 4 |
| HEC 2% + Honey (3:2) | 6 | 33% | 1 |
| HEC 2% + 30% sucrose | 5 | 40% | 1 |
| 15ml tube with FTA-card and sponge | **341** | **12%** | **8** |
| Cage | **236** | **2%** | **4** |
| 2% HEC + Honey (3:2) | 118 | 2% | 2 |
| 2% HEC + 30% sucrose | 118 | 2% | 2 |
| BG | **105** | **23%** | **4** |
| 2% HEC + 30% sucrose | 51 | 20% | 2 |
| 2% HEC + Honey (3:2) | 54 | 26% | 2 |
| 15ml tube with FTA-card | **940** | **42%** | **36** |
| Cage | **469** | **23%** | **14** |
| 2% HEC + Honey (3:2) | 240 | 22% | 7 |
| 2% HEC + 30% sucrose | 229 | 24% | 7 |
| BG | **471** | **54%** | **22** |
| 2% HEC + 30% sucrose | 140 | 46% | 7 |
| 2% HEC + Honey (3:2) | 331 | 58% | 15 |
| Total | **2250** | **35%** | **83** |

**Table S1. Sugar feeding tests performed with *Aedes albopictus* using different solutions and delivery systems.**

**S1b. WNV-1 detection from FTA-card collected at different time points.**

WNV-1 CT 678 strain was released on FTA cards at 3.25x10^5^ PFU/ml diluted 1:50 (20µl of seed + 980 µl of dilution buffer) directly spotted on the substrate to obtain a final viral load of 32.5 PFU (5µl + 15µl dilution buffer). Feeders with infected FTA cards were placed in BG traps and collected at different time points (7-, 10- and 15-days post deployment), testing 2 traps outdoors and 1 trap indoors for each time point. RNA extraction and detection of WNV-1 from collected FTA-cards was performed as described in Materials and Methods. All viral RNA from FTA-card was successfully amplified at all time points (Tab. S2, Fig. 1 and 2).

| **7 days** | | **10 days** | | **15 days** | |
| --- | --- | --- | --- | --- | --- |
| Tm mean | Ct mean | Tm mean | Ct mean | Tm mean | Ct mean |
| 85,12 | 31,28 | 85,10 | 31,21 | 85,15 | 31,57 |
| 85,19 | 31,89 | 85,12 | 31,36 | 85,22 | 31,34 |
| 85,07 | 31,48 | 85,14 | 31,19 | 85,12 | 30,78 |

**Table S2. Signal parameters of real-time PCR for** **WNV-1 extracted from FTA cards at different time points .** Mean ct values (ct mean) and melting temperature (Tm) of tested samples.


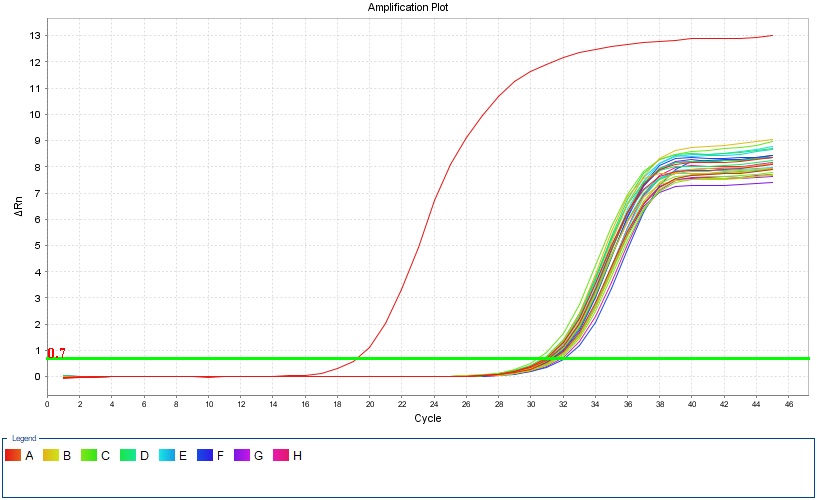


**Figure S1-** **Amplification curve of real-time PCR for** **WNV-1 extracted from FTA cards at different time points.** In red is reported the positive control (field strain of USUV RNA extracted from a cell culture, 2.5* 10^5 TCID50/ml), the other curves represent tested samples and negative control (NTC: No Template Control). All samples were tested in triplicate wells.


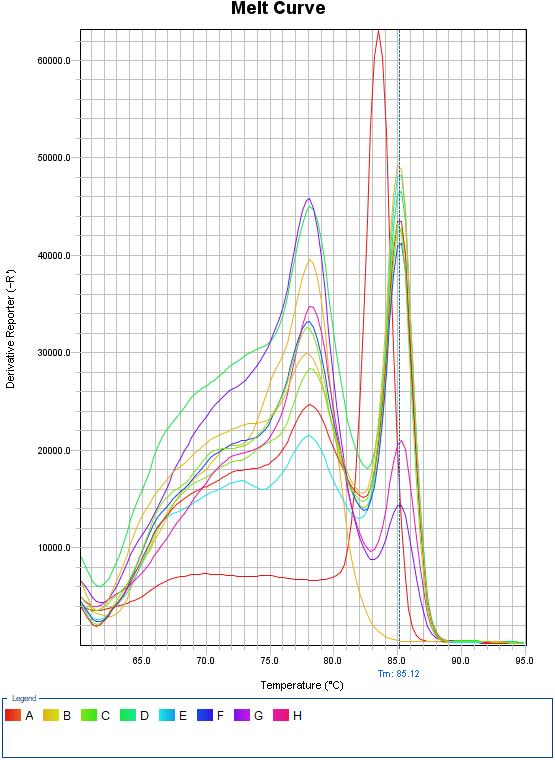

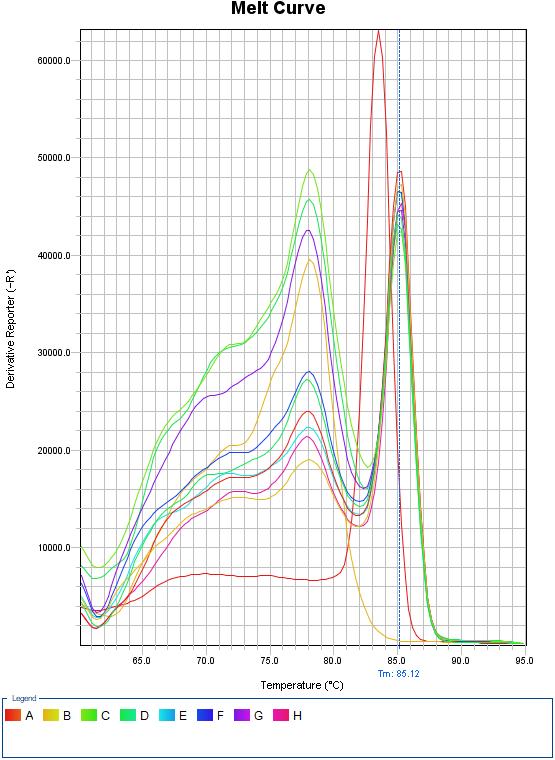

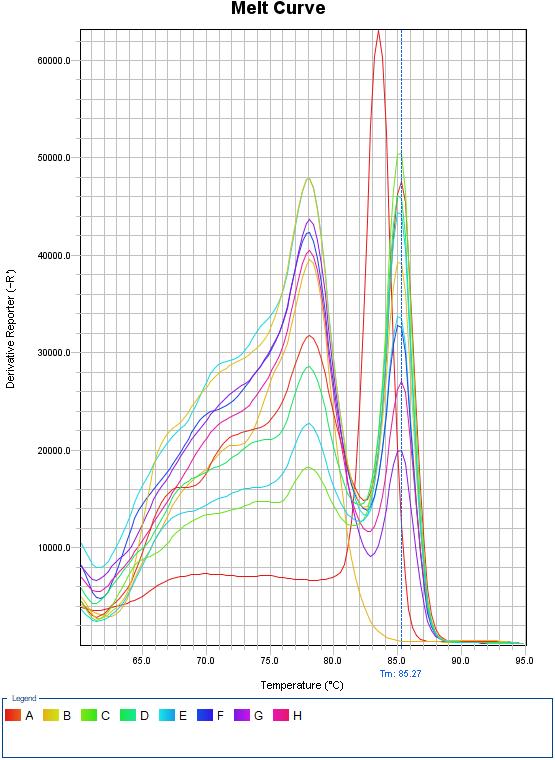


7 days

15 days

10 days

*

*

*

**Figure S2 - Melt curve plot.** In red is reported the positive control (field strain of USUV RNA extracted from a cell culture, 2.5* 10^5 TCID50/ml), the other curves represent tested samples and negative control (NTC: No Template Control, marked as *). All samples were tested in triplicate wells.
