# Supplementary material for: A modified BG-Sentinel trap equipped with FTA card as a novel tool for mosquito-borne disease surveillance: a field test for flavivirus detection
Source: Sci Rep. 2023 Aug 8;13:12840. doi: 10.1038/s41598-023-39857-1 (PMC10409816; doi:10.1038/s41598-023-39857-1)
Supplement: Supplementary file 2 — Supplementary Information 2. [file 41598_2023_39857_MOESM2_ESM.docx]

**Supplementary file 2­**

**S2a. GLM models of Species abundance and diversity**

| variable | description |
| --- | --- |
| **MOSQUITO_TOT** | Total number of mosquitoes of all species counted in each collection |
| **CPFEM** | Number of *Culex pipiens* females counted in each collection |
| **OCFEM** | Number of *Ochelrotatus caspius* females counted in each collection |
| **AAFEM** | Number of *Aedes albopictus* females counted in each collection |
| **CMFEM** | Number of *Culex modestus* females counted in each collection |
| **ANMAFEM** | Number of *Anopheles maculipennis s.l.* females counted in each collection |
| **SH** | Species diversity calculated as Shannon diversity index |
| **Method** | Variable for either of the two trap types (BG, CDC) |
| BG | BG-Sentinel 2 trap = (Intercept) |
| CDC | CDC trap |
| **Site** | Variable for either of the ten collection site (Badia Polesine, Caorle, Ceneselli, Erbé, Ficarolo, Jesolo, Minerbe, Nogarole Rocca, Oppeano, Villa Bartolomea) |
| **julian** | Collection day reported as julian day |
| **total_time** | Working time calculated in hours per each collection |

ABUNDANCE

# 1 glm negative binomial of all species
## Call:
## glm.nb(formula = MOSQUITO_TOT ~ Method + Site + julian + offset(log(total_time)),
## data = df1, init.theta = 2.181469549, link = log)
##
## Deviance Residuals:
## Min 1Q Median 3Q Max
## -3.5876 -0.9037 -0.2243 0.3814 1.9185
##
## Coefficients:
## Estimate Std. Error z value Pr(>|z|)
## (Intercept) 9.561127 1.059475 9.024 < 2e-16 ***
## MethodCDC 0.485001 0.152122 3.188 0.00143 **
## SiteCaorle -0.207698 0.340504 -0.610 0.54188
## SiteCeneselli -0.581275 0.341001 -1.705 0.08827 .
## SiteErbé 0.523406 0.340146 1.539 0.12386
## SiteFicarolo 0.726698 0.339842 2.138 0.03249 *
## SiteJesolo 0.057103 0.340252 0.168 0.86672
## SiteMinerbe 0.486717 0.340932 1.428 0.15341
## SiteNogarole Rocca 0.332223 0.340277 0.976 0.32890
## SiteOppeano 0.635049 0.340085 1.867 0.06186 .
## SiteVillabartolomea -0.225839 0.341481 -0.661 0.50839
## julian -0.034914 0.004902 -7.122 1.06e-12 ***
## ---
## Signif. codes: 0 '***' 0.001 '**' 0.01 '*' 0.05 '.' 0.1 ' ' 1
##
## (Dispersion parameter for Negative Binomial(2.1815) family taken to be 1)
##
## Null deviance: 183.406 on 79 degrees of freedom
## Residual deviance: 86.342 on 68 degrees of freedom
## AIC: 1072.9
##
## Number of Fisher Scoring iterations: 1
##
##
## Theta: 2.181
## Std. Err.: 0.329
##
## 2 x log-likelihood: -1046.908

## # R2 for Generalized Linear Regression
## Nagelkerke's R2: 0.782

#confidence interval

#CDC trap estimate=1.6 95% CI=1.21-2.19

exp(0.49)

## [1] 1.632316

exp(0.485001+1.96*0.152122)

## [1] 2.188377

exp(0.485001-1.96*0.152122)

## [1] 1.205437

#julian day estimate=0.96 95% CI=0.96-0.98
exp(-0.034914)

## [1] 0.9656885

exp(-0.034914+1.96*0.004902)

## [1] 0.9750114

exp(-0.034914-1.96*0.004902)

## [1] 0.9564546

#site Ficarolo estimate=2.07 95% CI=1.06-4.03
exp(0.726698)

## [1] 2.06824

exp(0.726698+1.96*0.339842)

## [1] 4.02606

exp(0.726698-1.96*0.339842)

## [1] 1.062482

#2 glm negative binomial Culex pipiens

## Call:
## glm.nb(formula = CPFEM ~ Method + Site + julian + offset(log(total_time)),
## data = df1, init.theta = 1.975442277, link = log)
##
## Deviance Residuals:
## Min 1Q Median 3Q Max
## -3.4399 -0.8956 -0.1699 0.4422 1.6737
##
## Coefficients:
## Estimate Std. Error z value Pr(>|z|)
## (Intercept) 11.277155 1.116831 10.097 < 2e-16 ***
## MethodCDC 0.764592 0.160335 4.769 1.85e-06 ***
## SiteCaorle -0.678882 0.360158 -1.885 0.0594 .
## SiteCeneselli -0.540186 0.359820 -1.501 0.1333
## SiteErbé 0.432898 0.358509 1.207 0.2272
## SiteFicarolo 0.686088 0.358005 1.916 0.0553 .
## SiteJesolo 0.209435 0.358460 0.584 0.5590
## SiteMinerbe 0.777047 0.358882 2.165 0.0304 *
## SiteNogarole Rocca 0.314761 0.358658 0.878 0.3802
## SiteOppeano 0.811182 0.358169 2.265 0.0235 *
## SiteVillabartolomea 0.029992 0.359571 0.083 0.9335
## julian -0.045840 0.005171 -8.865 < 2e-16 ***
## ---
## Signif. codes: 0 '***' 0.001 '**' 0.01 '*' 0.05 '.' 0.1 ' ' 1
##
## (Dispersion parameter for Negative Binomial(1.9754) family taken to be 1)
##
## Null deviance: 235.202 on 79 degrees of freedom
## Residual deviance: 87.115 on 68 degrees of freedom
## AIC: 1013.7
##
## Number of Fisher Scoring iterations: 1
##
##
## Theta: 1.975
## Std. Err.: 0.300
##
## 2 x log-likelihood: -987.683

## # R2 for Generalized Linear Regression
## Nagelkerke's R2: 0.890

#confidence interval

#CDC estimate= 2.16 95% CI=1.57-2.94
exp(0.764592)

## [1] 2.159766

exp(0.764592+1.96*0.160335)

## [1] 2.941291

exp(0.764592-1.96*0.160335)

## [1] 1.568838

#site Minerbe estimate= 2.18 95% CI=1.08-4.39
exp(0.777047)

## [1] 2.17504

exp(0.777047+1.96*0.358882)

## [1] 4.394948

exp(0.777047-1.96*0.358882)

## [1] 1.076417

#site Oppeano estimate= 2.25 95% CI=1.12-4.54
exp(0.811182)

## [1] 2.250567

exp(0.811182+1.96*0.358169)

## [1] 4.541209

exp(0.811182-1.96*0.358169)

## [1] 1.115353

#julian day estimate 0.96 95% CI=0.95-0.96

exp(-0.045840)

## [1] 0.9551948

exp(-0.045840+1.96*0.005171)

## [1] 0.9649251

exp(-0.045840-1.96*0.005171)

## [1] 0.9455626

#3 glm negative binomial Ochleroratus caspius

## Call:
## glm.nb(formula = OCFEM ~ Method + Site + julian + offset(log(total_time)),
## data = df1, init.theta = 0.8613203172, link = log)
##
## Deviance Residuals:
## Min 1Q Median 3Q Max
## -2.2549 -0.9646 -0.4874 0.2033 1.8948
##
## Coefficients:
## Estimate Std. Error z value Pr(>|z|)
## (Intercept) 3.829584 1.779625 2.152 0.0314 *
## MethodCDC -0.225473 0.256711 -0.878 0.3798
## SiteCaorle 0.390938 0.542707 0.720 0.4713
## SiteCeneselli -0.687715 0.545739 -1.260 0.2076
## SiteErbé 0.547170 0.542824 1.008 0.3135
## SiteFicarolo 0.754214 0.542197 1.391 0.1642
## SiteJesolo -1.043598 0.547812 -1.905 0.0568 .
## SiteMinerbe -4.961085 0.797123 -6.224 4.85e-10 ***
## SiteNogarole Rocca -2.212472 0.564241 -3.921 8.81e-05 ***
## SiteOppeano -1.140885 0.549194 -2.077 0.0378 *
## SiteVillabartolomea -2.301388 0.567118 -4.058 4.95e-05 ***
## julian -0.013529 0.008254 -1.639 0.1012
## ---
## Signif. codes: 0 '***' 0.001 '**' 0.01 '*' 0.05 '.' 0.1 ' ' 1
##
## (Dispersion parameter for Negative Binomial(0.8613) family taken to be 1)
##
## Null deviance: 190.951 on 79 degrees of freedom
## Residual deviance: 89.301 on 68 degrees of freedom
## AIC: 656.3
##
## Number of Fisher Scoring iterations: 1
##
##
## Theta: 0.861
## Std. Err.: 0.141
##
## 2 x log-likelihood: -630.303

## # R2 for Generalized Linear Regression
## Nagelkerke's R2: 0.792

#confidence interval

#site Minerbe estimate= 0.007 95% CI=0.001-0.03
exp(-4.961085)

## [1] 0.007005323

exp(-4.961085+1.96*0.797123)

## [1] 0.03341588

exp(-4.961085-1.96*0.797123)

## [1] 0.001468599

#site Nogarole Rocca estimate= 0.11 95% CI=0.04-0.33
exp(-2.212472)

## [1] 0.1094298

exp(-2.212472+1.96*0.564241)

## [1] 0.3306947

exp(-2.212472-1.96*0.564241)

## [1] 0.03621129

#site Oppeano estimate= 0.32 95% CI=0.11-0.94
exp(-1.140885)

## [1] 0.3195361

exp(-1.140885+1.96*0.549194)

## [1] 0.9375692

exp(-1.140885-1.96*0.549194)

## [1] 0.1089022

#site Villabartolomea estimate= 0.10 95% CI= 0.03-0.30
exp(-2.301388)

## [1] 0.1001198

exp(-2.301388+1.96*0.567118)

## [1] 0.3042709

exp(-2.301388-1.96*0.567118 )

## [1] 0.03294423

# 4 glm negative binomial Aedes albopictus

## Call:
## glm.nb(formula = AAFEM ~ Method + Site + julian + offset(log(total_time)),
## data = df1, init.theta = 1.140184114, link = log)
##
## Deviance Residuals:
## Min 1Q Median 3Q Max
## -2.5117 -0.9268 -0.4895 0.2633 2.1117
##
## Coefficients:
## Estimate Std. Error z value Pr(>|z|)
## (Intercept) -1.909795 1.699866 -1.123 0.261226
## MethodCDC -1.997896 0.252101 -7.925 2.28e-15 ***
## SiteCaorle -0.057691 0.511608 -0.113 0.910218
## SiteCeneselli -1.582522 0.564022 -2.806 0.005019 **
## SiteErbé -0.637267 0.526678 -1.210 0.226289
## SiteFicarolo 0.784728 0.499557 1.571 0.116218
## SiteJesolo -0.058478 0.511624 -0.114 0.909001
## SiteMinerbe -0.289584 0.520405 -0.556 0.577898
## SiteNogarole Rocca -2.224388 0.615297 -3.615 0.000300 ***
## SiteOppeano 0.996665 0.498375 2.000 0.045519 *
## SiteVillabartolomea -2.270303 0.632459 -3.590 0.000331 ***
## julian 0.009494 0.007891 1.203 0.228963
## ---
## Signif. codes: 0 '***' 0.001 '**' 0.01 '*' 0.05 '.' 0.1 ' ' 1
##
## (Dispersion parameter for Negative Binomial(1.1402) family taken to be 1)
##
## Null deviance: 218.259 on 79 degrees of freedom
## Residual deviance: 86.937 on 68 degrees of freedom
## AIC: 471.07
##
## Number of Fisher Scoring iterations: 1
##
##
## Theta: 1.140
## Std. Err.: 0.235
##
## 2 x log-likelihood: -445.073

## # R2 for Generalized Linear Regression
## Nagelkerke's R2: 0.863

#confidence interval

#CDC estimate=0.14 95% CI=0.08-0.22
exp(-1.997896)

## [1] 0.1356203

exp(-1.997896+1.96*0.252101)

## [1] 0.2222888

exp(-1.997896-1.96*0.252101)

## [1] 0.08274316

#site Ceneselli estimate=0.21 95% CI=0.067-0.62
exp(-1.582522)

## [1] 0.2054563

exp(-1.582522+1.96*0.564022)

## [1] 0.6206184

exp(-1.582522-1.96*0.564022)

## [1] 0.06801649

#site Nogarole Rocca estimate=0.11 95% CI= 0.03-0.36
exp(-2.224388)

## [1] 0.1081336

exp(-2.224388+1.96*0.615297)

## [1] 0.3611702

exp(-2.224388-1.96*0.615297)

## [1] 0.03237496

#site Oppeano estimate=2.71 95% CI=1.02-7.20
exp(0.996665)

## [1] 2.709231

exp(0.996665+1.96*0.498375)

## [1] 7.195674

exp(0.996665-1.96*0.498375)

## [1] 1.020048

#site Villabartolomea estimate=0.10 95% CI=0.03-0.35
exp(-2.270303)

## [1] 0.1032809

exp(-2.270303+1.96*0.632459)

## [1] 0.3567631

exp(-2.270303-1.96*0.632459)

## [1] 0.02989923

# 5 glm negative binomial Culex modestus

## Call:
## glm.nb(formula = CMFEM ~ Method + Site + julian + offset(log(total_time)),
## data = df1, init.theta = 0.7993704232, link = log)
##
## Deviance Residuals:
## Min 1Q Median 3Q Max
## -2.126 -0.567 -0.368 0.000 1.478
##
## Coefficients:
## Estimate Std. Error z value Pr(>|z|)
## (Intercept) -9.390e+00 3.017e+00 -3.113 0.001855 **
## MethodCDC -3.457e-01 4.258e-01 -0.812 0.416818
## SiteCaorle -1.716e+00 1.282e+00 -1.338 0.180819
## SiteCeneselli -1.652e+00 1.244e+00 -1.328 0.184080
## SiteErbé 2.792e+00 7.325e-01 3.812 0.000138 ***
## SiteFicarolo -3.652e+01 2.373e+07 0.000 0.999999
## SiteJesolo -1.643e+00 1.252e+00 -1.313 0.189342
## SiteMinerbe -3.629e+01 2.373e+07 0.000 0.999999
## SiteNogarole Rocca 3.189e+00 7.295e-01 4.372 1.23e-05 ***
## SiteOppeano 1.064e+00 7.738e-01 1.375 0.169081
## SiteVillabartolomea -1.379e+00 1.255e+00 -1.099 0.271630
## julian 2.842e-02 1.382e-02 2.056 0.039735 *
## ---
## Signif. codes: 0 '***' 0.001 '**' 0.01 '*' 0.05 '.' 0.1 ' ' 1
##
## (Dispersion parameter for Negative Binomial(0.7994) family taken to be 1)
##
## Null deviance: 211.475 on 79 degrees of freedom
## Residual deviance: 46.198 on 68 degrees of freedom
## AIC: 205.99
##
## Number of Fisher Scoring iterations: 1
##
##
## Theta: 0.799
## Std. Err.: 0.272
##
## 2 x log-likelihood: -179.989

## # R2 for Generalized Linear Regression
## Nagelkerke's R2: 0.940

#confidence interval

#site Erbè estimate=16.31 95% CI=3.88-68.56
exp(2.792e+00)

## [1] 16.31361

exp(2.792e+00+1.96*7.325e-01)

## [1] 68.55936

exp(2.792e+00-1.96*7.325e-01)

## [1] 3.881804

#Nogarole Rocca estimate=24.26 95% CI=5.81-101.37
exp(3.189e+00)

## [1] 24.26415

exp(3.189e+00+1.96*7.295e-01)

## [1] 101.3743

exp(3.189e+00-1.96*7.295e-01)

## [1] 5.807673

#julian day estimate=1.03 95% CI=1.04-1.07
exp(2.842e-02)

## [1] 1.028828

exp(2.842e-02*1.96+1.382e-02)

## [1] 1.071997

exp(2.842e-02*1.96-1.382e-02)

## [1] 1.042773

# 6 glm negative binomial Anopheles maculipennis s.l.

## Call:
## glm.nb(formula = ANMAFEM ~ Method + Site + julian + offset(log(total_time)),
## data = df1, init.theta = 1.188647409, link = log)
##
## Deviance Residuals:
## Min 1Q Median 3Q Max
## -2.0742 -0.7829 -0.3588 0.0000 2.1651
##
## Coefficients:
## Estimate Std. Error z value Pr(>|z|)
## (Intercept) -2.246e+00 2.366e+00 -0.949 0.342452
## MethodCDC -8.065e-01 3.356e-01 -2.403 0.016239 *
## SiteCaorle 1.159e+00 9.831e-01 1.179 0.238392
## SiteCeneselli -3.547e+01 2.373e+07 0.000 0.999999
## SiteErbé 3.654e+00 8.930e-01 4.092 4.27e-05 ***
## SiteFicarolo 1.541e+00 9.505e-01 1.621 0.104995
## SiteJesolo -5.711e-01 1.343e+00 -0.425 0.670614
## SiteMinerbe 1.394e-01 1.131e+00 0.123 0.901854
## SiteNogarole Rocca 5.670e+00 8.847e-01 6.409 1.46e-10 ***
## SiteOppeano 3.133e+00 8.994e-01 3.483 0.000495 ***
## SiteVillabartolomea -3.537e+01 2.373e+07 0.000 0.999999
## julian -9.493e-03 1.066e-02 -0.890 0.373427
## ---
## Signif. codes: 0 '***' 0.001 '**' 0.01 '*' 0.05 '.' 0.1 ' ' 1
##
## (Dispersion parameter for Negative Binomial(1.1886) family taken to be 1)
##
## Null deviance: 325.942 on 79 degrees of freedom
## Residual deviance: 56.086 on 68 degrees of freedom
## AIC: 259.9
##
## Number of Fisher Scoring iterations: 1
##
##
## Theta: 1.189
## Std. Err.: 0.385
##
## 2 x log-likelihood: -233.901

## # R2 for Generalized Linear Regression
## Nagelkerke's R2: 0.982

#confidence interval

# CDC estimate=0.45 95% CI= 0.23-0.86
exp(-8.065e-01)

## [1] 0.4464178

exp(-8.065e-01+1.96*3.356e-01)

## [1] 0.8618069

exp(-8.065e-01-1.96*3.356e-01)

## [1] 0.2312454

#site Erbé estimate=0.47 95% CI=6.71-222.36
exp(3.654e+00)

## [1] 38.62887

exp(3.654e+00+1.96*8.930e-01)

## [1] 222.3561

exp(3.654e+00-1.96*8.930e-01)

## [1] 6.710812

#site Nogarole Rocca estimate=290.03 95% CI=119.74-1642.56
exp(5.670e+00)

## [1] 290.0345

exp(5.670e+00+1.96*8.847e-01)

## [1] 1642.561

exp(5.670e+00-8.847e-01)

## [1] 119.7373

#site Oppeano estimate=22.94 95% CI=3.94-22.94
exp(3.133e+00)

## [1] 22.9427

exp(3.133e+00+1.96*8.994e-01)

## [1] 133.7302

exp(3.133e+00-1.96*8.994e-01)

## [1] 3.936043

#SHANNON INDEX DIVERSITY (SH)

## Call:
## glm(formula = SH ~ Method + Site + julian + offset(log(total_time)),
## data = df1)
##
## Deviance Residuals:
## Min 1Q Median 3Q Max
## -0.44468 -0.13981 -0.01058 0.12441 0.66324
##
## Coefficients:
## Estimate Std. Error t value Pr(>|t|)
## (Intercept) -4.055678 0.341329 -11.882 < 2e-16 ***
## MethodCDC -0.332147 0.049009 -6.777 3.56e-09 ***
## SiteCaorle 0.038260 0.109587 0.349 0.72807
## SiteCeneselli -0.234869 0.109587 -2.143 0.03568 *
## SiteErbé -0.017472 0.109632 -0.159 0.87385
## SiteFicarolo -0.092412 0.109587 -0.843 0.40203
## SiteJesolo -0.201384 0.109587 -1.838 0.07048 .
## SiteMinerbe -0.475784 0.109930 -4.328 5.06e-05 ***
## SiteNogarole Rocca 0.027851 0.109632 0.254 0.80023
## SiteOppeano -0.143466 0.109632 -1.309 0.19507
## SiteVillabartolomea -0.337753 0.109930 -3.072 0.00305 **
## julian 0.009159 0.001579 5.802 1.88e-07 ***
## ---
## Signif. codes: 0 '***' 0.001 '**' 0.01 '*' 0.05 '.' 0.1 ' ' 1
##
## (Dispersion parameter for gaussian family taken to be 0.04803707)
##
## Null deviance: 9.7392 on 79 degrees of freedom
## Residual deviance: 3.2665 on 68 degrees of freedom
## AIC: -2.8339
##
## Number of Fisher Scoring iterations: 2

1 - mod.SH$deviance/mod.SH$null.deviance

## [1] 0.6645996

#confidence interval

# CDC estimate=-0.33 95% CI=-0.24- -0.43
(-0.332147+1.96*0.049009)

## [1] -0.2360894

(-0.332147-1.96*0.049009)

## [1] -0.4282046

#site Censelli estimate=-0.23 95% CI=-0.02- -0.44
-0.234869

## [1] -0.234869

-0.234869+1.96*0.109587

## [1] -0.02007848

-0.234869-1.96*0.109587

## [1] -0.4496595

#site Minerbe estimate=-0.48 95% CI=-0.26- -0.69

## [1] -0.475784

-0.475784+1.96*0.109930

## [1] -0.2603212

-0.475784-1.96*0.109930

## [1] -0.6912468

#site Villabartolomea estimate=-0.34 95% CI=-0.12- -0.55
-0.337753

## [1] -0.337753

-0.337753+1.96*0.109930

## [1] -0.1222902

-0.337753-1.96*0.109930

## [1] -0.5532158

#julian day estimate=0.01 95% CI= 0.006-0.01
0.009159

## [1] 0.009159

0.009159+1.96*0.001579

## [1] 0.01225384

0.009159-1.96*0.001579

## [1] 0.00606416

S2b. Estimate of the proportion of infected individuals in pooled samples (Maximum likelihood estimate, MLE)

| variable | description |
| --- | --- |
| **Pos** | Positive pool reported as 0 or 1. The MLE was estimated for each virus (USUV or WNV) |
| **PoolSize** | Number of mosquitoes in a single pool |
| **NumPools** | Number of pools (in each observation only 1 pool) |
| **Method** | Trap types (BG or CDC) |
| **Zone** | Collection site in 2019 sampling (Badia Polesine, Caorle, Ceneselli, Erbé, Ficarolo, Jesolo, Minerbe, Nogarole Rocca, Oppeano, Villa Bartolomea) |
| **Week** | Collection week of 2021 sampling (30, 31, 32, 33, 34) |

#MLE 2019 USUV

## Estimation of Binomial Proportion for Pooled Data
##
## Call: pooledBin(x = Pos ~ m(PoolSize) + (NumPools) | Method * Zone, data = usuv.2019cdcbg, pt.method = "mle")
##
## Point estimator : Maximum Likelihood
## CI method : Skew-Corrected Score (Gart)
## Confidence coefficient : 95%
##
## Method Zone P Lower Upper Scale N
## 1 CDC Minerbe 0.0007850244 1.385374e-04 0.0025423500 1 2648
## 2 CDC Villabartolomea 0.0000000000 0.000000e+00 0.0023583995 1 1625
## 3 CDC Ficarolo 0.0000000000 0.000000e+00 0.0020413297 1 1878
## 4 CDC Ceneselli 0.0000000000 0.000000e+00 0.0074905388 1 509
## 5 CDC Badia Polesine 0.0007619425 4.371076e-05 0.0035632161 1 1328
## 6 CDC Nogarole Rocca 0.0000000000 0.000000e+00 0.0021402775 1 1791
## 7 CDC Erbé 0.0013424964 2.345933e-04 0.0043485020 1 1591
## 8 CDC Oppeano 0.0020748618 7.655843e-04 0.0045225717 1 2630
## 9 BG Minerbe 0.0010164957 2.647013e-04 0.0027265705 1 3090
## 10 BG Villabartolomea 0.0000000000 0.000000e+00 0.0026866327 1 1426
## 11 BG Ceneselli 0.0000000000 0.000000e+00 0.0051367289 1 744
## 12 BG Badia Polesine 0.0000000000 0.000000e+00 0.0021237123 1 1805
## 13 BG Ficarolo 0.0001939515 1.108662e-05 0.0009318663 1 5189
## 14 BG Nogarole Rocca 0.0000000000 0.000000e+00 0.0021915609 1 1749
## 15 BG Erbé 0.0011057803 2.885289e-04 0.0029587820 1 2833
## 16 BG Oppeano 0.0019677503 5.066111e-04 0.0052773748 1 1669
## NumPools NumPosPools
## 1 31 2
## 2 23 0
## 3 25 0
## 4 12 0
## 5 21 1
## 6 26 0
## 7 27 2
## 8 36 5
## 9 44 3
## 10 32 0
## 11 26 0
## 12 36 0
## 13 67 1
## 14 38 0
## 15 47 3
## 16 34 3

#MLE WNV 2019

## Estimation of Binomial Proportion for Pooled Data
##
## Call: pooledBin(x = Pos ~ m(PoolSize) + (NumPools) | Method * Zone, data = wnv.2019cdcbg, pt.method = "mle")
##
## Point estimator : Maximum Likelihood
## CI method : Skew-Corrected Score (Gart)
## Confidence coefficient : 95%
##
## Method Zone P Lower Upper Scale N
## 1 CDC Minerbe 0.0003825777 2.182358e-05 0.0018240424 1 2648
## 2 CDC Villabartolomea 0.0000000000 0.000000e+00 0.0023583995 1 1625
## 3 CDC Ficarolo 0.0005444585 3.088934e-05 0.0025934332 1 1878
## 4 CDC Ceneselli 0.0021849381 1.180053e-04 0.0102998781 1 509
## 5 CDC Badia Polesine 0.0000000000 0.000000e+00 0.0028843214 1 1328
## 6 CDC Nogarole Rocca 0.0005717017 3.241482e-05 0.0027207516 1 1791
## 7 CDC Erbé 0.0000000000 0.000000e+00 0.0024086776 1 1591
## 8 CDC Oppeano 0.0003875703 2.199588e-05 0.0018577834 1 2630
## 9 BG Minerbe 0.0006677813 1.180290e-04 0.0021676610 1 3090
## 10 BG Villabartolomea 0.0000000000 0.000000e+00 0.0026866327 1 1426
## 11 BG Ceneselli 0.0014273072 7.898860e-05 0.0068303774 1 744
## 12 BG Badia Polesine 0.0005697924 3.214742e-05 0.0027350132 1 1805
## 13 BG Ficarolo 0.0000000000 0.000000e+00 0.0007397605 1 5189
## 14 BG Nogarole Rocca 0.0000000000 0.000000e+00 0.0021915609 1 1749
## 15 BG Erbé 0.0003588421 2.038957e-05 0.0017229043 1 2833
## 16 BG Oppeano 0.0000000000 0.000000e+00 0.0022963675 1 1669
## NumPools NumPosPools
## 1 31 1
## 2 23 0
## 3 25 1
## 4 12 1
## 5 21 0
## 6 26 1
## 7 27 0
## 8 36 1
## 9 44 2
## 10 32 0
## 11 26 1
## 12 36 1
## 13 67 0
## 14 38 0
## 15 47 1
## 16 34 0

#MLE WNV 2021

## Estimation of Binomial Proportion for Pooled Data
##
## Call: pooledBin(x = Pos ~ m(PoolSize) + (NumPools) | Method * Week, data = wnv.dat2021, pt.method = "mle")
##
## Point estimator : Maximum Likelihood
## CI method : Skew-Corrected Score (Gart)
## Confidence coefficient : 95%
##
## Method Week P Lower Upper Scale N NumPools
## 1 BG 30 0.000000000 0.000000e+00 0.003093357 1 1238 28
## 2 CDC 31 0.000000000 0.000000e+00 0.016641113 1 227 4
## 3 BG 31 0.000000000 0.000000e+00 0.005396509 1 708 19
## 4 CDC 32 0.000000000 0.000000e+00 0.020670623 1 182 4
## 5 BG 32 0.000000000 0.000000e+00 0.003748344 1 1021 18
## 6 CDC 33 0.000000000 0.000000e+00 0.023590177 1 159 3
## 7 BG 33 0.003006547 7.604236e-04 0.008131256 1 1150 24
## 8 CDC 34 0.000000000 0.000000e+00 0.014956537 1 253 4
## 9 BG 34 0.001192597 6.602819e-05 0.005724795 1 889 25
## NumPosPools
## 1 0
## 2 0
## 3 0
## 4 0
## 5 0
## 6 0
## 7 3
## 8 0
## 9 1

#MLE USUV 2021

## Estimation of Binomial Proportion for Pooled Data
##
## Call: pooledBin(x = Pos ~ m(PoolSize) + (NumPools) | Method * Week, data = usuv.dat2021, pt.method = "mle")
##
## Point estimator : Maximum Likelihood
## CI method : Skew-Corrected Score (Gart)
## Confidence coefficient : 95%
##
## Method Week P Lower Upper Scale N NumPools
## 1 BG 30 0.002716563 0.0006966319 0.007255662 1 1238 28
## 2 CDC 31 0.000000000 0.0000000000 0.016641113 1 227 4
## 3 BG 31 0.002899804 0.0005316969 0.008756517 1 708 19
## 4 CDC 32 0.000000000 0.0000000000 0.020670623 1 182 4
## 5 BG 32 0.004822089 0.0015027620 0.011520885 1 1021 18
## 6 CDC 33 0.009864689 0.0004246799 0.049062541 1 159 3
## 7 BG 33 0.004244450 0.0013142535 0.010272930 1 1150 24
## 8 CDC 34 0.005016889 0.0002506355 0.023309276 1 253 4
## 9 BG 34 0.006863407 0.0025116704 0.014729535 1 889 25
## NumPosPools
## 1 3
## 2 0
## 3 2
## 4 0
## 5 4
## 6 1
## 7 4
## 8 1
## 9 5
